# School water, sanitation, and hygiene inequalities: a bane of sustainable development goal six in Nigeria

**DOI:** 10.17269/s41997-022-00633-9

**Published:** 2022-04-11

**Authors:** Ojima Zechariah Wada, David Bamidele Olawade, Eunice Oluwafolakemi Oladeji, Aminat Opeyemi Amusa, Elizabeth Omoladun Oloruntoba

**Affiliations:** 1 Division of Sustainable Development, College of Science and Engineering, Hamad Bin Khalifa University, Qatar Foundation, Doha, Qatar; 2 Department of Environmental Health Sciences, Faculty of Public Health, University of Ibadan, Ibadan, Nigeria; 3 Department of Public Health, University of Southern Mississippi, Hattiesburg, MS USA; 4 Roseberry Park Hospital, Tees, Esk and Wear Valleys, Middlesbrough, United Kingdom

**Keywords:** Water, Sanitation, Hygiene, School WASH, WASH inequalities, Open defecation, Eau, assainissement, hygiène, WASH dans les écoles, inégalités WASH, défécation en plein air

## Abstract

**Objectives:**

The importance of school water, sanitation, and hygiene (WASH) in achieving the Sustainable Development Goal targets 6.1 and 6.2 in developing countries cannot be overemphasized. However, widespread WASH inequalities remain an impediment to achieving the targets by 2030. Hence, this study was conducted to examine current school-WASH disparities among public and private schools in a low-income Nigerian community using mixed methods.

**Methods:**

The cross-sectional survey utilized multi-stage sampling to select 400 students from five public and five private schools in Akinyele, Ibadan. Semi-structured questionnaires and observational checklists were used to obtain data. Inferential statistics were measured at a 95% confidence interval. Independent variables like the students’ sociodemographic characteristics, school type, and available WASH facilities were associated with dependent variables like respondents’ hand hygiene and sanitation practices and WASH-associated knowledge and attitude to examine existing inequalities.

**Results:**

Classifying the available WASH facilities based on the WHO/UNICEF Joint Monitoring Programme, none of the public schools provided any sanitation and hygiene service, while all the private schools provided both services. Furthermore, the private-school students had significantly better WASH knowledge (*p*<0.001; *Ƞ*^2^_p_=0.152) and attitude (*p*<0.001; *Ƞ*^2^_p_=0.036) compared with the public-school students. Also, a significantly higher portion of public-school students practiced open defecation at school (*p*<0.001; odds ratio (OR)=7.4; confidence interval (CI)=4.1–13.5) and at home (*p*<0.001; OR=7.8; CI=3.7–16.7).

**Conclusion:**

WASH disparities among socioeconomic groups remain a persistent challenge. Sole reliance on the Government to narrow the inequalities has persistently proven unfruitful. There is a need to empower local community stakeholders to facilitate sustainable school-WASH interventions.

## Résumé

**Objectifs:**

On ne saurait trop insister sur l’importance de l’eau, de l’assainissement et de l’hygiène (WASH) dans les écoles pour atteindre les cibles 6,1 et 6,2 des objectifs de développement durable dans les pays en développement. Toutefois, les inégalités généralisées en matière de WASH demeurent un obstacle à la réalisation des objectifs d’ici à 2030. Par conséquent, cette étude a été menée pour examiner les disparités actuelles entre les écoles publiques et privées dans une communauté nigériane à faible revenu en utilisant des méthodes mixtes.

**Méthodes:**

L'enquête transversale a utilisé un échantillonnage à plusieurs étapes pour sélectionner 400 élèves de cinq écoles publiques et cinq écoles privées à Akinyele, Ibadan. Des questionnaires semi-structurés et des listes de contrôle observationnelles ont été utilisés pour obtenir des données. Les statistiques inférentielles ont été mesurées à un intervalle de confiance de 95 %. Des variables indépendantes comme les caractéristiques sociodémographiques des élèves, le type d’école et les installations de WASH disponibles ont été associées à des variables dépendantes comme les pratiques d’hygiène des mains et d’assainissement des répondants et les connaissances et l’attitude associées au WASH pour examiner les inégalités existantes.

**Résultats:**

Si l’on classe les installations WASH disponibles sur la base du Programme commun OMS/UNICEF de suivi, aucune des écoles publiques ne fournit les services d’assainissement et d’hygiène, alors que toutes les écoles privées fournissent ces deux services. En outre, les élèves des écoles privées avaient une connaissance nettement meilleure de WASH (*p*<0,001; *Ƞ*^2^_p_=0,152) et attitude (*p*<0,001; *Ƞ*^2^_p_=0,036) par rapport aux élèves des écoles publiques. De plus, une proportion significativement plus élevée d’élèves des écoles publiques pratiquaient la défécation en plein air à l’école (*p*<0,001; rapport de cotes (RC)=7,4; intervalle de confiance (IC)=4,1–13,5) et à domicile (*p*<0,001; OR=7,8; IC=3,7–16,7).

**Conclusion:**

Les disparités WASH entre les groupes socio-économiques demeurent un problème persistant. Le fait de compter uniquement sur le gouvernement pour réduire les inégalités s’est toujours avéré infructueux. Il est nécessaire de donner aux intervenants communautaires locaux les moyens de faciliter des interventions WASH durables en milieu scolaire.

## Introduction

Water, sanitation, and hygiene (WASH) are three interdependent pillars of preventive health. In Nigeria, poor sanitation and hygiene practices are highly prevalent; evidence of this is seen from the country’s recent status as the world’s open defecation capital (Idowu, 2019). However, with a majority of Nigeria’s citizens within the educational age bracket, prioritizing school-based intervention is integral for instilling healthy sanitation and hygiene knowledge, attitudes, and practices (KAP) into the future generation (UNICEF, 2012). Children spend a significant portion of their childhood and adolescent years in the school environment. With an average duration of over 6 h per day, this sums up to over 1100 h spent every year in the school vicinity (Micaiah, 2014). This implies that the state of the school environment has a significant impact on the students’ well-being, habits, and perceptions. The WHO/UNICEF Joint Monitoring Programme for Water Supply, Sanitation, and Hygiene has not only posited the importance of school WASH on the students’ well-being but has also recommended the provision of basic WASH facilities. Breaking down the terms, basic drinking water service simply implies the availability of a functional improved water source (free from contamination), and basic sanitation service implies the presence of usable, single-sex, and improved toilet facilities (where faeces is separated from human contact), while basic hygiene service implies the presence of functional handwash facilities with soap and running water present (WHO/UNICEF, 2018).

The paucity of basic school-WASH services in Nigeria has been revealed to be prevalent, and to contribute significantly to poor sanitation and hygiene practices of youths and adolescents (Egbinola & Amanambu, 2015; Wada et al., 2020; Wada & Oloruntoba, 2021). Moreover, the situation is worsened due to the widespread social inequalities that exist in the Nigerian WASH sector. In 2017, the World Bank estimated that around 90% of rural Nigerians defecate in the open, and indicated that 51% of rural communities did not have access to improved water (World Bank Group, 2017). The rural/urban disparities are mostly a result of the differences in wealth quantiles. Urban areas tend to have a higher number of wealthier households and stronger economic power. Hence, the political will for providing basic WASH and social infrastructure in rural areas tends to be relatively lower (Ojima et al., 2020; Sinharoy et al., 2019). Another study that monitored the progress made in WASH in sub-Saharan Africa revealed that rural poor households were 29 times less likely to access improved water and 25 times less likely to access improved sanitation facilities when compared to the urban poor (Armah et al., 2018). Moreover, wealthier households in these rural areas have better WASH services when compared to other households (Chasekwa et al., 2018). In addition, unhealthy sanitation and hygiene practices among Nigerian youths and adolescents have also been associated with inadequate knowledge and negative attitude towards proper hygiene and sanitation (Azuogu et al., 2016; UNICEF, 2015). This makes it paramount to look beyond just assessing for the availability of adequate WASH facilities when conducting school-WASH surveys. The students’ associated knowledge and attitudes are also important variables to examine.

The WASH disparities that exist between socio-economic groups make the achievement of Sustainable Development Goal targets 6.1 (universal and equitable access to safe and affordable drinking water) and 6.2 (access to adequate and equitable sanitation and hygiene for all and an end to open defecation, paying special attention to the needs of women and girls and those in vulnerable situations) by 2030 quite improbable (WHO/UNICEF, 2018). To date, data on school-WASH inequalities in Nigeria is sparse, making it difficult to assess the extent of damage. Hence, this study seeks to assess the WASH facilities present among schools in a Nigerian low-income community and to examine the students’ sanitation and hygiene KAP. The study also identifies WASH inequalities that exist between public and private schools and across the students’ socio-economic class. With the paucity of local data pertaining to school-WASH inequalities, findings from this survey provide information in building a roadmap to reduce the disease and economic burden associated with the prevalent unhealthy sanitation and hygiene practices in Nigeria.

## Methods

### Study area

The survey was conducted in Akinyele Local Government Area (LGA) Ibadan, Nigeria. The LGA is one of the eleven LGAs in Ibadan metropolis, bordered by Afijo LGA, Lagelu LGA, Ido LGA, and Ibadan North LGA to the north, east, west, and south respectively. Its land coverage spans over 500 km^2^, with an estimated population of about 240,000 as of 2010. Furthermore, the peri-urban LGA consists of 12 wards, with a significant portion of the locals dependent on agriculture as a source of livelihood. The LGA consists of numerous rural low-income households and has 33 public senior secondary schools and around 30 private schools. This area was selected because of its mixed socioeconomic group which is key in identifying spatial inequalities. Also, the location has had recurring cases of cholera outbreaks over the past years, which resulted in the Nigeria Centre for Disease Control making recommendations for sustainable WASH interventions (Adekunle, 2018). Hence, output from this research could be a testament to the area’s WASH resilience.

### Study design

A cross-sectional study design was employed for this survey. Data were obtained via a pre-tested semi-structured questionnaire and an observational checklist. The pre-test was conducted among secondary school students in Ibadan North LGA, a neighbouring LGA. By using Cronbach’s alpha, the internal consistency of the questionnaire was estimated to be 0.849. A mixed-methods approach was taken in order to have two objective independent assessments—one from the researchers via the observational checklist and the other from the respondents via questionnaire. This design was chosen in order to understand the current state of school WASH across the entire LGA. Results from this research would be valuable in designing future interventional or longitudinal studies in the study area.

### Study population

The study population consisted of senior secondary school students in Akinyele LGA. Senior classes comprising Senior Students (SS) 1, 2, and 3 were selected because the students would have spent at least 3 years in their respective schools and would also be expected to have formed their WASH-related attitudes and practice. Senior high schools were also selected because these students are the bridge between adolescence and adulthood. With poor WASH practices prevalent among Nigerian adults, having an insight into their WASH-related KAP at adolescence would provide information on how to break unhealthy habits.

The sample size of 351 students was estimated using the Leslie Kish formula, based on a 35.4% prevalence of poor sanitation practice as reported in a recent study (Wada et al., 2020), at 95% level of confidence and 5% precision. To account for non-response and to increase the statistical power, the sample size was increased to 400 students. Eventually, a 99.5% response rate was achieved.

### Sampling technique

A multi-stage sampling technique was used to select the students. First, 5 wards were randomly selected from the 11 wards in the LGA via balloting. Second, in each ward, one public and one private secondary school were selected via simple random sampling. A total of 10 schools were selected. Third, via stratified sampling, students were proportionally selected from each school based on the schools’ population size. For confidentiality, the private schools were renamed PS 1, PS 2, PS 3, PS 4, and PS 5, while the government schools were renamed GS 1, GS 2, GS 3, GS 4, and GS 5.

### Data collection instrument and procedure

The semi-structured questionnaire was divided into 4 sections: sociodemographic, WASH-related knowledge assessment, WASH-related attitude assessment, and sanitation and hygiene practice. Most of the questions were adopted from the WHO/UNICEF Joint Monitoring Programme (JMP) Core questions and indicators for monitoring WASH in Schools in the Sustainable Development Goals and recent surveys by Wada et al. (Wada et al., 2020, 2021a; WHO/UNICEF, 2018). The observational checklist was also adopted from the WHO/UNICEF JMP school WASH guide, UNICEF school WASH monitoring package, and Nigerian school sanitation policy (FMOE, 2006; UNICEF, 2011; WHO/UNICEF, 2018). Data collection spanned from May 2019 to September 2019.

The questionnaire copies were administered by five trained research assistants (RAs). With the aid of the RAs, the students were monitored during the process to ensure there was no external influence from their peers or teachers. The RAs also vetted the questionnaires to ensure the students filled them appropriately. The environmental assessment was handled solely by the principal investigator.

### Data management and analysis

Data obtained were entered into Microsoft Excel, cleaned, and then exported to SPSS version 20 and JASP 0.14.1.0 for statistical analysis. The WASH facilities available in the school were categorized based on the WHO/UNICEF JMP Ladders for school sanitation (WHO/UNICEF, 2018). Also, the respondents’ WASH knowledge and attitude were categorized into good and poor knowledge and good, fair, and poor attitude, respectively. Knowledge scores ranged from 0 to 12, and respondents who possessed good knowledge obtained scores between 8 and 12, while those with poor knowledge had scores below 8. Furthermore, attitude levels ranged from 0 to 20. Students with positive attitude obtained scores between 0 and 5, and those with fair attitude had scores between 6 and 10, while students with negative attitude had scores over 10.

Descriptive statistics for the students’ WASH KAP were presented via measures of frequency and proportion. Inferential statistics were performed at 95% confidence interval via bivariate analysis like one-way ANOVA and chi-square test for independence, and multivariate analysis like logistic regression. ANOVA was performed to assess for significant differences between the respondents’ mean knowledge and attitude scores and their sociodemographic characteristics. In cases where variances were unequal (i.e., significant Levene’s test), ANOVA with Welch was used for the analysis and Games-Howell post hoc test was used in place of Tukey’s standard post hoc test. The effect size of significant associations was measured using partial eta (*Ƞ*^2^_p_). Chi-square was used to identify variables significantly associated with open defecation practice and poor handwash practice. The effect size for significant associations was measured using Cramer’s *V*. Finally, logistic regression was used to identify the predictors of the sanitation and hygiene practice of interest.

## Results

### Respondents’ sociodemographic characteristics

Details of this can be found in Table 1. The students had an average age of 15.65±1.67 years, ranging from 11 to 21 years. More students attended the government schools because they are very affordable to the common man; however, these schools are mostly underfunded. Considering the private school students, a majority of their parents had attained tertiary education and were working as professionals in the formal sector (health, banking, and education), while a majority of the public school students’ parents attained below tertiary level of education and worked informal jobs (trading, farming, and auto mechanics). Over 60% of the respondents also revealed that at least one under-5 child was present in their households (1.80+2.13 children per household).

**Table 1:** Respondents’ sociodemographic characteristics

Characteristics	Frequency (*N*=398)	Percentage (%)
Sex		
Male	164	41.3
Female	233	58.7
Age of respondents (years)		
11–16	288	72.4
17–21	110	27.6
Type of school		
Public school	307	77.1
Private school	91	22.9
Mother’s highest level of education		
Primary	50	12.8
Secondary	216	54.9
Tertiary	108	27.2
No formal education	22	5.1
Mother’s occupation		
Trader	274	68.8
Food seller	20	5.0
Civil servant	49	12.3
Housewife	14	3.5
Formal sector*	41	10.3
Father’s highest level of education		
Primary	40	10.0
Secondary	180	45.2
Tertiary	147	37.0
No formal education	3	0.8
Father’s occupation		
Trader	120	30.1
Mechanic	74	18.5
Civil servant	88	22.1
Farmer	29	7.2
Formal sector*	88	22.1
Ethnic group		
Yoruba	347	87.2
Hausa	14	3.4
Igbo	19	4.8
Igala, Idoma, Tiv, Egun	18	4.5
Religion		
Christianity	206	51.8
Islam	189	47.5
Traditional/atheist	3	0.8
Households with under-5 children	200	63.7

*Formal sector consists of professionals like health workers, bankers, and academics

### Disparities in available school sanitation and hygiene facilities

Inspection of the available school WASH facilities revealed that all the public schools provided no sanitation and hygiene service, while 80% provided no water service and the remaining 20% provided limited service. Forty percent of the public schools had no toilet facilities available, while the remainder had facilities that were not functional at the time of the survey. Furthermore, none of the private schools provided drinking water service, 80% provided basic sanitation service, and 100% provided limited hygiene service. Table 2 sheds light on the WASH services provided by each school. Moreover, assessing the schools for their solid waste management revealed that 100% of the public schools practiced open burning, while 60% of the private schools practiced the same. The remaining private schools had their waste handled by local waste collectors.

**Table 2:** JMP classification of available WASH facilities

School	Water	Sanitation	Hygiene
GS 1	No service	No service^c^	No service
GS 2	No service	No service^d^	No service
GS 3	No service	No service^e^	No service
GS 4	No service	No service^d^	No service
GS 5	Limited^a^	No service^c^	No service
PS 1	No service	Basic (pour flush)	Limited (no soap)
PS 2	No service	Basic (pour flush)	Limited (no soap)
PS 3	No service	Basic (pour flush)	Limited (no soap)
PS 4	No service	Basic (pour flush)	Limited (no soap)
PS 5	No service	Limited^b^	Limited (no soap)

*GS* government school, *PS* private school

^a^Presence of an electric pump borehole with no water due to absence of power supply

^b^Pour flush toilet available, but common use

^c^No toilet facilities available

^d^Toilet facilities available but not functional

^e^Toilet facilities available but not accessible to the students

### Respondents’ WASH-related knowledge and attitude

Generally, a significant portion of knowledge gaps identified were associated with understanding the process and importance of hand washing and the extent to which open defecation practices is dangerous. The average knowledge score of the respondents was estimated to be 7.5±2.14 (range of 0 to 12), with 55.8% possessing good knowledge (range of 8 to 12) and the remainder possessing poor knowledge (range of 0 to 7).

Furthermore, the major attitudinal concerns identified were the students’ perceptions towards using the school toilets, open defecation, and routinely engaging in healthy hand hygiene practice with soap and water. In addition, the average attitude score for the students was 6.75+3.36 (range of 0 to 20), with 35.2% having positive attitude (range of 0 to 5), 49.7% fair attitude (range of 6 to 10), and 15.1% negative attitude (range of 11 to 20). Tables 3 and 4 provide the frequencies and proportions of the respondents’ responses to each knowledge and attitude question, respectively.

**Table 3:** Proportion of respondents’ responses to knowledge questions

Knowledge statement	Frequency (*N*=398)	Proportion (%)
Correct order of handwashing process		
Wet your hands then lather with soap then scrub then rinse then dry	224	56.3
Reason soap is used for handwashing		
To remove germs from the hands	330	82.9
Hand hygiene is most effective with soap and water	249	62.6
Reported diseases proper handwashing can prevent		
Diarrhoea	153	38.4
Malaria	126	31.7
Gonorrhea	57	14.3
HIV/AIDS	38	9.5
Moments to practice handwashing		
Before eating	151	37.9
After using the toilet	272	68.3
Most appropriate place to defecate		
Toilet	342	85.9
Open defecation spots	51	12.8
Open defecation in school could lead to spread of disease	326	83.4
Pour-flush toilet is more hygienic than pit/bucket latrines and shot-put	275	69.1
Faeces (poop) contain millions of germs	96	24.1
Single-sex toilets are ideal at school as opposed to common-use toilets	341	85.7
Cholera is a waterborne disease	226	56.8

**Table 4:** Proportion of respondents’ responses to attitudinal questions

Attitude questions	SA (%)	A (%)	UD (%)	D (%)	SD (%)
I feel comfortable using the school toilets	39 (9.8)	71 (17.8)	24 (6.1)	147 (36.9)	117 (29.4)
There are days I’d rather stay back at home due to the lack of access to functional water, toilet and hygiene facilities at school	43 (10.8)	134 (33.7)	43 (10.8)	103 (25.9)	75 (18.8)
I’d rather defecate in the open than use the school toilet	64 (16.1)	96 (24.1)	41 (10.3)	112 (28.1)	85 (21.4)
I feel open defecation has a negative impact on the environment	112 (28.1)	137 (34.4)	43 (10.5)	67 (16.8)	40 (10.1)
I prefer keeping my nails long	34 (8.5)	69 (17.3)	55 (13.8)	154 (38.7)	86 (21.6)
I feel it is important to wash my hands with soap and water after defecating	213 (53.5)	151 (37.9)	11 (2.8)	13 (3.3)	10 (2.5)
I feel it is necessary to wash my hands with soap and water before eating	165 (41.5)	166 (41.7)	36 (9.1)	20 (5.0)	11 (2.8)
It is easier and more convenient to wash my hands with just water	37 (9.3)	110 (27.6)	52 (13.1)	148 (37.2)	51 (12.8)
Colourless/clear water without taste or odour from any source is fit for drinking	135 (33.9)	102 (25.6)	32 (8.0)	79 (19.8)	50 (12.6)
I feel public burning of waste is an appropriate method of waste disposal	56 (14.1)	110 (27.6)	51 (12.9)	105 (26.4)	76 (19.1)
Disposal of waste into a water body or gutter is an appropriate method of waste disposal	45 (11.3)	66 (16.6)	44 (11.0)	111 (27.9)	132 (33.2)
Recycling waste is the most appropriate method of waste disposal	137 (34.4)	140 (35.2)	41 (10.3)	55 (13.8)	25 (6.3)

*SA* strongly agree, *A* agree, *UD* undecided, *D* disagree, *SD* strongly disagree

### Socioeconomic inequalities associated with respondents’ knowledge and attitude

Table 5 provides details of the bivariate analysis. There were statistically significant associations between the respondents’ knowledge and school type (*p*<0.001), class level (*p*<0.001), mother’s level of education (*p*=0.004), mother’s occupation (*p*=0.004), father’s level of education (*p*<0.001), and father’s occupation (*p*=0.002). However, only school type had a large effect size (partial Eta of 0.152), while the others had small to medium effect sizes. Respondents from private schools had a significantly higher knowledge score compared with public school students. Also, students from SS 1 and SS 3 had a significantly higher knowledge score than students in SS 2. Parents of respondents who had attained tertiary education and worked in the formal sector as professionals had significantly higher knowledge levels than their counterparts.

**Table 5:** Association between respondents’ knowledge/attitude and their sociodemographic characteristics

Variables		Respondents’ knowledge						Respondents’ attitude				
		Sum of squares	df	Mean square	*F*	*p* value	*Ƞ*^2^_p_	Sum of squares	Mean square	*F*	*p* value	*Ƞ*^2^_p_
All schools	Between groups	368.78	9	40.96	10.93	<0.001*	0.202	310.29	34.48	3.20	<0.001*	0.069
	Within groups	1454.72	388	3.75				4176.08	10.76			
School type	Between groups	277.24	1	277.24	71.00	<0.001*	0.152	161.17	161.17	14.76	<0.001*	0.036
	Within groups	1546.26	396	3.91				4325.20	10.92			
Class level	Between groups	88.75	2	44.38	10.10	<0.001*	0.049	23.99	11.99	1.06	0.350	0.005
	Within groups	1734.75	395	4.39				4462.39	11.30			
Gender	Between groups	2.03	1	2.03	0.442	0.507	0.001	4.23	4.23	0.37	0.541	9.44e^-4^
	Within groups	1821.47	396	4.60				44.82	11.32			
Mother’s education	Between groups	61.08	3	20.36	4.55	0.004*	0.033	38.32	12.77	0.82	0.489	0.009
	Within groups	1762.42	394	4.47				4448.05	62.87			
Mother’s occupation	Between groups	70.34	4	17.59	3.94	0.004*	0.039	119.23	29.81	2.68	0.031*	0.027
	Within groups	1753.16	393	4.46				4367.14	11.11			
Father’s education	Between groups	153.50	3	51.17	12.04	<0.001*	0.084	101.99	33.99	3.05	0.029*	0.023
	Within groups	1669.75	393	4.25				4383.82	11.16			
Father’s occupation	Between groups	74.12	4	18.53	4.23	0.002*	0.042	56.14	4	14.04	0.297	0.013
	Within groups	1697.90	388	4.38				4418.81	388	11.39		

Furthermore, there were statistically significant associations between the respondents’ attitude and school type (*p*<0.001), mother’s occupation (*p*=0.031), and father’s level of education (*p*=0.029). All the effect sizes were between small and medium. Public school students had a significantly higher attitude score (indicating poorer attitude) than private school students. Also, students whose mothers worked as professionals and those whose fathers had attained tertiary education had significantly lower attitudinal scores compared with their counterparts.

### Respondents’ WASH practice

An overview of the respondents’ practices is presented in Table 6. Just around 30% of the students reported practicing handwashing with soap and water while at school. The major reasons identified for skipping handwashing were absence of water and forgetfulness. Less than 50% of the students routinely used the school toilets for urination and defecation, while 51.1% of the students admitted to practicing open defecation at school. The common places used for such practice were the bush, fields, and uncompleted buildings. Furthermore, 50% of the students also admitted to practicing open defecation at home, the most commonly used spots being the bush, fields, and uncompleted buildings. In addition, over 70% of the students practiced open burning of solid waste while at school.

**Table 6:** Respondents’ WASH practice at school and home sanitation practice

WASH practice at school	Frequency (*N*=398)	Proportion (%)
Availability of soap and water for hand hygiene at school	143	35.9
How hand hygiene is practiced at school		
With only water	184	47.7
With soap and water	117	30.3
Zero hand hygiene practiced at school	81	21.0
At random	29	1.0
Moments hands were washed (*multiple responses*)		
After break time	154	38.9
Before eating	227	57.0
After using the toilet	313	78.6
Other moments	33	8.3
Major reasons for skipping handwashing		
Forgetfulness	127	33.1
No attributed importance	61	15.9
No water	140	36.5
No handwash station at school	52	13.5
Routine places urination was practiced		
School toilet	182	45.7
In the bush, field, stream, behind toilet	138	34.7
I do not ease myself while at school	78	19.6
Routine places defecation was practiced		
School toilet	187	47.0
In the bush, field, stream, behind toilet	103	25.9
I do not ease myself while at school	108	27.1
Alternative defecation spots used by students (51.1%)		
Bush/fields	122	30.7
Uncompleted building	40	10.1
Behind the toilet	25	6.3
Stream, shot-put	16	4.0
No alternative	195	48.9
Accessibility to school toilets round-the-clock	243	61.1
Availability of classes that teach sanitation and hygiene	254	63.8
Availability of water within the school toilet	223	58.7
Availability of cleaners appointed to clean toilets	139	34.9
Practice of open burning for waste management	299	75.1
Home sanitation practice		
Toilet used at home		
Pit latrine with slab	74	18.6
Pit latrine without slab	31	7.7
Water closet	283	71.2
No toilet/latrine	10	2.5
Shared toilet facilities with neighbours	50	12.6
Routine places defecation was practiced		
Toilet	357	89.8
Bush, field, stream, uncompleted building	41	10.2
Alternative defecation spots at home (50.0%)		
Bush/fields	59	14.8
Uncompleted building	52	13.1
Behind the toilet	29	7.3
Stream, shot-put	59	14.8
No alternative spot	199	50.0

### Socioeconomic inequalities associated with open defecation practice and hand hygiene practice

Using bivariate analysis, open defecation practice was revealed to be significantly associated with school type (*p*<0.001), gender (*p*<0.001), mother’s highest level of education (*p*=0.041), father’s highest level of education (*p*=0.001), father’s occupation (*p*=0.002), respondents’ knowledge category (*p*<0.001), respondents’ attitude category (*p*<0.001), accessibility to school toilets (*p*=0.014), availability of a toilet cleaner (*p*<0.001), availability of water in the toilet (*p*=0.001), and open defecation practice at home (*p*<0.001). Moreover, the variables open defecation practice at home, school type, father’s level of education, respondents’ knowledge, respondents’ attitude, and availability of toilet cleaner had the highest effect sizes. In addition, public school students were 7.3 times more likely than private school students to engage in open defecation practice, while male students were 1.99 times more likely than female students to do so. Respondents who practiced open defecation at home were 6.13 times more likely to practice the act in school than those who did not practice this at home. Students from schools where toilet cleaners were employed and students with good knowledge were 3 times less likely than their counterparts to practice open defecation. In addition, students whose parents had attained tertiary education and whose fathers worked in formal sectors were less likely to engage in such practice. Only 14.6% of respondents whose mothers are professionals practiced open defecation as compared with 50%, 42.9%, 40.0%, and 35.8% open defecation rate among children of housewives, civil servants, food sellers, and traders, respectively. Also, only 22.1% of students whose fathers had attained tertiary education practiced open defecation at home as compared with 43.3% of students whose fathers had no formal education. Moreover, only 17.4% of students whose fathers were professionals practiced open defecation at home as compared with 50% of respondents whose fathers were farmers.

Moreover, hand hygiene practice was significantly associated with gender (*p*=0.049), availability of toilet cleaner (*p*<0.001), availability of water in the school toilet (*p*=0.005), students’ comfortability using the school toilet (*p*<0.001), and practice of open defecation at home (*p*=0.042). However, the effect sizes were between small and medium. Students from schools with toilet cleaners were 2.8 times more likely to engage in healthy hand hygiene practice (with soap and water) than students from schools without cleaners. Schools that had water in toilet facilities increased the likelihood of their students engaging in healthy hand hygiene practice by 1.9 times. Also, male students and students who practiced open defecation at home had a 1.5 times lower likelihood of engaging in healthy hand hygiene practice at school.

Details of the associations are presented in Table 7.

**Table 7:** Factors associated with open defecation practice and healthy hand hygiene practice

Variables	Open defecation practice						Healthy hand hygiene practice					
	Count (proportion)		*χ*^2^	*p*-value	Log odds ratio and CI	Cramer’s *V*	Count (proportion)		*χ*^2^	*p*-value	Log odds ratio and CI	Cramer’s *V*
**School**	***Yes***	***No***					***Yes***	***No***				
Public	187 (60.9%)	120 (39.1%)	52.74	<0.001*	1.989 1.402 to 2.575	0.364	84 (27.4%)	223 (72.6%)	2.68	0.102	−0.412 −0.908 to 0.083	0.082
Private	16 (17.6%)	75 (82.4%)					33 (36.3%)	58 (63.7%)				
**Gender**	***Yes***	***No***					***Yes***	***No***				
Male	100 (61.0%)	64 (39.0%)	11.10	<0.001*	0.687 0.280 to 1.093	0.167	57 (34.8%)	107 (65.2%)	3.86	0.049*	0.435 −0.025 to 0.870	0.098
Female	103 (44.0%)	131 (56.0%)					60 (25.6%)	174 (74.4%)				
**MHLE**	***Yes***	***No***					***Yes***	***No***				
None	9 (45.0%)	11 (55.0%)	8.23	0.041*	-	0.143g	6 (30.0%)	14 (70.0%)	0.20	0.978	-	0.035g
Primary	24 (47.1%)	27 (52.9%)					16 (31.4%)	35 (68.6%)				
Secondary	126 (57.3%)	94 (42.7%)					65 (29.5%)	155 (70.5%)				
Tertiary	44 (41.1%)	63 (58.9%)					30 (28.0%)	77 (72.0%)				
**FHLE**	***Yes***	***No***					***Yes***	***No***				
None	14 (46.7%)	16 (53.3%)	16.22	0.001*	-	0.292g	8 (26.7%)	22 (73.3%)	1.32	0.725	-	0.094^g^
Primary	24 (61.5%)	15 (38.5%)					12 (30.8%)	27 (69.2%)				
Secondary	109 (59.6%)	74 (40.4%)					58 (31.7%)	125 (68.3%)				
Tertiary	56 (38.6%)	89 (61.4%)					38 (26.2%)	107 (73.8%)				
**MO**	***Yes***	***No***					***Yes***	***No***				
Formal	40 (44.4%)	50 (55.6%)	2.08	0.149	−0.347 −0.819 to 0.125	0.072	26 (28.9%)	64 (71.1%)	0.014	0.904	−0.031 −0.550 to 0.486	0.006
Informal	163 (53.1%)	144 (46.9%)					91 (29.5%)	217 (70.5%)				
**FO**	***Yes***	***No***					***Yes***	***No***				
Formal	72 (42.1%)	99 (57.9%)	9.80	0.002*	−0.641 −3.344 to −0.237	0.002	48 (27.9%)	124 (72.1%)	0.32	0.569	−0.127 −0.564 to 0.308	0.029
Informal	131 (58.0%)	95 (42.0%)					69 (30.5%)	157 (69.5%)				
**Age (years)**	***Yes***	***No***					***Yes***	***No***				
11–16	138 (48.1%)	149 (51.9%)	3.86	0.050	−0.445 −0.889 to 0.001	0.099	82 (28.5%)	206 (71.5%)	0.43	0.512	−0.159 −0.635 to 0.317	0.033
17–21	65 (59.1%)	45 (40.9%)					35 (31.8%)	75 (68.2%)				
**Knowledge**	***Yes***	***No***					***Yes***	***No***				
Poor	116 (65.9%)	60 (34.1%)	28.05	<0.001*	1.099 0.687 to 1.511	0.265	51 (29.0%)	125 (71.0%)	0.03	0.870	−0.008 −0.471 to 0.398	0.008
Good							66 (29.7%)	156 (70.3%)				
**Attitude**	***Yes***	***No***					***Yes***	***No***				
Positive	51 (36.4%)	89 (63.6%)	23.45	<0.001*	-	0.243	47 (33.6%)	93 (66.4%)	1.87	0.393	-	0.069
Fair	109 (55.1%)	89 (44.9%)					53 (26.8%)	145 (73.2%)				
Negative	43 (71.7%)	17 (28.22%)					17 (28.3%)	43 (71.7%)				
**Toilet access**	***Yes***	***No***					***Yes***	***No***				
Yes	122 (46.1%)	131 (53.9%)	6.03	0.014*	−0.509 −0.916 to −0.937	0.123	73 (30.0%)	170 (70.0%)	0.13	0.724	0.080 −0.364 to 0.524	0.018
No	91 (58.7%)	64 (41.3%)					44 (28.4%)	111 (71.6%)				
**Toilet cleaner**	***Yes***	***No***					***Yes***	***No***				
Present	46 (33.1%)	93 (66.9%)	27.42	<0.001*	−1.135 −1.568 to −0.703	0.262	61 (43.9%)	78 (56.1%)	21.6	<0.001*	1.042 0.595 to 1.489	0.233
Absent	157 (60.6%)	102 (39.4%)					56 (21.6%)	203 (78.4%)				
**OD at home**	***Yes***	***No***					***Yes***	***No***				
Yes	109 (77.9%)	31 (22.1%)	62.31	<0.001*	1.814 1.341 to 2.287	0.396	50 (35.7%)	90 (64.3%)	4.15	0.042*	0.460 0.016 to 0.904	0.102
No	94 (36.4%)	164 (63.6%)					67 (26.0%)	191 (74.0%)				
**Toilet water**	***Yes***	***No***					***Yes***	***No***				
Present	101 (44.1%)	128 (55.9%)	10.28	0.001*	−0.657 −1.061 to −0.253	0.161	80 (34.9%)	149 (65.1%)	7.97	0.005*	0.650 0.195 to 1.105	0.141
Absent	102 (60.4%)	67 (39.6%)					37 (21.9%)	132 (78.1%)				

*CI* confidence interval, *MHLE* mother’s highest level of education, *FHLE* father’s highest level of education, *MO* mother’s occupation, *FO* father’s occupation, *OD* open defecation

^g^Signifies ordinal gamma was used to measure effect size

*Signifies association statistically significant at *p*<0.05.

### Predictors of open defecation and handwash practice

Using multivariate analysis, the variables that significantly predicted open defecation practice were attitude score, school type, gender, and practice of open defecation at home. The regression model revealed that school type was the strongest predictor variable, followed by practice of open defecation at home, attitude score, and then gender. Private school students were 2.1 times less likely to practice open defecation at school, and students who did not practice open defecation at home were 2 times less likely to practice open defecation at school, while female students were 1.4 times less likely. The likelihood of open defecation practice also reduced as the respondents’ attitude improved. This model had a sensitivity of 71.8%, implying it can fairly accurately predict the students’ open defecation practice.

Furthermore, the variables that significantly predicted hand hygiene practice were availability of water in school toilets, comfortability using school toilets, availability of toilet cleaners, and practice of open defecation at home. The strongest predictors in descending order were availability of toilet janitors, comfortability using school toilets, availability of water in toilets, and practice of open defecation at home. Students at schools with no janitors were 2.15 less likely to engage in healthy hand hygiene practice, while students uncomfortable using school toilets were 2.65 times less likely. This model had a sensitivity of 94.7% implying it can highly predict poor hand hygiene practice among students in the study area. Table 8 provides details of the multivariate analysis.

**Table 8:** Predictors of open defecation practice and hand hygiene practice

Coefficients	Estimate	Standard error	Odds ratio	*p*-value	Wald statistics
*No open defecation practice (Nagelkerke R*^*2*^*= 0.335; AIC = 446.47; BIC = 466.40; p<0.001)*					
Intercept	0.93	0.29	2.53	0.001	10.17
Attitude score	0.12	0.04	0.89	<0.001	11.25
School type (private)	0.75	0.16	0.47	<0.001	22.22
Gender (female)	0.34	0.12	0.71	0.004	8.24
OD at home (no)	0.69	0.13	0.50	<0.001	28.75
*Poor hand hygiene (Nagelkerke R*^*2*^*= 0.165; AIC = 443.21; BIC = 463.14; p<0.001)*					
Intercept	1.09	0.32	0.34	<0.001	11.63
Toilet water absent	0.64	0.25	1.90	0.011	6.46
Uncomfortable using toilet	0.97	0.26	2.65	<0.001	14.21
Absence of toilet cleaners	1.15	0.25	2.15	<0.001	21.35
OD at home (no)	0.58	0.26	1.78	0.024	5.10

*OD open defecation

## Discussion

### Disparities in sanitation and hygiene facilities

This study revealed disparities between the public and private schools sampled, particularly with respect to sanitation and hygiene. This was contrary to similar school-WASH surveys conducted in Lagos, Nigeria, where conditions were similar across the board (Wada et al., 2020; Wada & Oloruntoba, 2021). Similar WASH-related public–private school disparities have also been reported by the JMP among sub-Saharan countries like Ghana, Mali, Togo, and Senegal (Steele, 2018). In addition, comparing the current study to another school WASH survey conducted in a neighbouring urban LGA by Egbinola and Amanambu, some disparities can also be noted. All the schools in the urban LGA had water sources available and around 11% provided basic hygiene service, while all the schools provided a form of sanitation facility (Egbinola & Amanambu, 2015). A similar urban–peri-urban trend was also reported by the last JMP report for school WASH (Steele, 2018). The absence of drinking water facilities in all the schools can be attributed to the lack of political will by the government and the predominance of affordable sachet water sold in most parts of Nigeria (Egbinola & Amanambu, 2015; Oloruntoba et al., 2021).

Furthermore, the absence of soap for hand hygiene in all the schools clearly depicts the poor handwash culture prevalent in the schools. This has also been reported in other Nigerian school WASH surveys, which recommended that handwash facilities need to be provided in order for the students to concretize healthy practice (Azuogu et al., 2016; Wada & Oloruntoba, 2021). It is counterintuitive for a society vulnerable to deadly diseases like Ebola, Lassa fever, and diarrhoea to perpetuate a poor handwash culture (Tambo et al., 2018). The emergence of COVID-19 has further reiterated the importance of hand hygiene in disease prevention (Gammon & Hunt, 2020). Concerted efforts by the government, civil societies, and NGOs are required to tackle this challenge via resource allocation, health promotion, and localized hand hygiene campaigns (Kumwenda, 2019).

### Inequalities in students’ WASH-associated knowledge, attitude, and practice

Disparities were also observed when comparing the KAP of public and private school students. Other socio-economic factors that affected respondents’ KAP were their parents’ level of education and occupation. Once again, the public school students were disadvantaged in all aspects. Moreover, the fact that a majority of their parents had not attained tertiary education and were working in the informal sector put them at further disadvantage. The negative impact of inequalities in income level and educational status on sanitation and hygiene practices has been corroborated by a number of studies (Morgan et al., 2017; Kumwenda, 2019; Wada & Oloruntoba, 2021). A nationwide survey utilizing national demographics also revealed that accessibility of Nigerians to improved sanitation facilities was dependent on socio-economic factors like highest educational level of household head and household wealth. Less educated and less wealthy households were more likely to engage in open defecation practice (Abubakar, 2017). In this study, three out of every five public school students engaged in open defecation, as compared with less than one out of every five private students. The overall 51.1% open defecation rate obtained in this study is higher than both the estimate obtained in a school-WASH survey conducted in a commercial centre (35.4%) and the national rate (24%) (Federal Government of Nigeria and UNICEF, 2016; Wada et al., 2020).

Furthermore, results from this study revealed how unhealthy WASH practice at school could influence students beyond the school environment (UNICEF, 2012). Over 77% of the respondents who practiced open defecation at school also engaged in the act while at home even though sanitation facilities were reported to be available in around 95% of the students’ homes. The low prevalence of healthy hand hygiene practice with soap and water at school (30.3%) was not surprising as all the schools lacked basic hygiene services. This was lower than the prevalence obtained in previous studies (Egbinola & Amanambu, 2015; Wada & Oloruntoba, 2021). The significant impact that presence/absence of toilet janitors had on the students’ sanitation and hygiene practice has also been reported in other studies (Wada et al., 2020; Shao et al., 2021). As school WASH interventions are being planned, besides providing adequate school-WASH facilities, it is imperative to make provision for janitors/cleaners to routinely maintain the facilities, and to incorporate long-term health promotion schemes targeted at addressing the identified KAP gaps (Berhe et al., 2020).

These findings speak to the work cut out for stakeholders in addressing the school-WASH infrastructure gap and WASH-knowledge gap of students which subsequently influences their attitude and practice of such. These stakeholders include parents, teachers and other school authorities, community leaders, and grassroots leaders who are closer to the rural households and their children. The efforts of community leaders in raising WASH awareness have direct and indirect effects on students in the community. The students learn directly from these awareness projects (multimedia jingles, easy-to-read flyers and billboards, and seminars) and also from watching their parents who have learned and are practicing healthy WASH behaviour. School authorities need to incorporate WASH knowledge into their curricula as this has been reported to improve WASH-related knowledge and attitudes and reduce water- and sanitation-related diseases among students (McMichael, 2019). By doing this and emphasizing the practice of WASH activities in the school, they will be reinforcing the effort of the parents back at home. The community stakeholders should also consider implementing future WASH interventions with locally resourced materials so the maintenance and sustainability of the project are assured. An example of such intervention was reported in a WASH survey among rural dwellers in southwestern Nigeria (Wada et al., 2021b). The government also needs to improve on tracking WASH-related data, especially in low-income and rural communities, in order to increase awareness of inequalities that exist in these communities and to assess the impact of WASH interventions carried out.

## Conclusion

It is alarming that none of the government schools provided basic sanitation service. This subjected around 60% of the affected students to poor practices like open defecation both at home and in school. Our study provides evidence that repeated acts eventually become habitual, which is why breaking the vicious cycle via school-based sanitation interventions is crucial. In addition, the poor hand hygiene culture displayed by all the schools puts the students at a great public health risk. With poor sanitation practices already prevalent, the absence of an enabling environment for healthy hand hygiene practice leaves the students vulnerable to hygiene-related diseases like diarrhoea and a reemergence of cholera outbreak episodes.

Future community-specific interventions targeting the identified school-WASH facilities and KAP gaps need to be implemented by local stakeholders like Parent–Teacher Associations, local school authorities, community leaders, and religious leaders. Sole reliance on the Government to provide basic school WASH infrastructure has persistently failed over the years. Hence, the local stakeholders must look within, and provide locally appropriate and cost-effective interventions using readily available materials. Local and international NGOs can work on enlightening local community stakeholders on simple, novel ways to produce soaps, handwash basins, and sanitation facilities.

## Contributions to knowledge

### What does this study add to existing knowledge?

- Prioritization of school WASH interventions is integral to achieving SDG 6 in Nigeria, as most of the country’s population are of school age.
- Data on school-WASH inequalities in Nigeria are sparse.
- This survey shows that public schools significantly lack sanitation and hygiene facilities as compared with private schools.
- The absence of such facilities also translated into poor associated knowledge, attitude, and practices.
- Harmful practices like open defecation were prevalent among public school students and became habitual as the majority also engaged in the act at home, even with the availability of private sanitation facilities.

### What are the key implications for public health interventions, practice, or policy?

- Providing adequate school-WASH facilities alone will not sufficiently curb the poor WASH practices prevalent in the schools as there is already an attitudinal and knowledge gap.
- Ensuring both public and private schools have equal access to basic WASH facilities will significantly reduce the sanitation- and hygiene-related morbidities and mortalities prevalent among the members of the low socioeconomic class, thereby reducing health inequalities by extension.
- Sole dependence on the Government to resolve school-WASH challenges has proven futile. Key agencies need to empower local stakeholders like the PTA, schoolteachers/principals, and community leaders in providing locally appropriate and cost-effective solutions.
